# The homeobox transcription factor MEIS2 is a regulator of cancer cell survival and IMiDs activity in Multiple Myeloma: modulation by Bromodomain and Extra-Terminal (BET) protein inhibitors

**DOI:** 10.1038/s41419-019-1562-9

**Published:** 2019-04-11

**Authors:** Maria Pia Abruzzese, Maria Teresa Bilotta, Cinzia Fionda, Alessandra Zingoni, Alessandra Soriani, Maria Teresa Petrucci, Maria Rosaria Ricciardi, Rosa Molfetta, Rossella Paolini, Angela Santoni, Marco Cippitelli

**Affiliations:** 1 Department of Molecular Medicine, Sapienza University of Rome, Rome, Italy; 2 Division of Hematology, Department of Cellular Biotechnologies and Hematology, Sapienza University of Rome, Rome, Italy; 3 Hematology, Department of Clinical and Molecular Medicine, Sapienza University of Rome, Rome, Italy; 4 Istituto Pasteur-Fondazione Cenci Bolognetti, Roma, RM Italy; 5 IRCCS, Neuromed, Pozzilli Italy

## Abstract

The transcription factor Myeloid Ecotropic Insertion Site 2 (MEIS2) has been identified as a cellular substrate of the E3-ubiquitin ligase complex CRL4-cereblon (^CRL4^CRBN) in crystal structure and by biochemical screen. Emerging evidence suggests that IMiDs can block MEIS2 from binding to CRBN facilitating the subsequent activation of a ^CRL4^CRBN^IMiD^-E3-ubiquitin ligase activity and proteasome-mediated degradation of critical substrates regulators of Multiple Myeloma (MM) cell survival and proliferation. Bromodomain and Extra-Terminal (BET) family of proteins are important epigenetic regulators involved in promoting gene expression of several oncogenes, and many studies have revealed important anticancer activities mediated by BET inhibitors (BETi) in hematologic malignancies including MM. Here, we investigated MEIS2 in MM, the role of this protein as a modulator of IMiDs activity and the ability of BETi to inhibit its expression. Our observations indicate that inhibition of MEIS2 in MM cells by RNA interference correlates with reduced growth, induction of apoptosis and enhanced efficacy of different anti-MM drugs. In addition, MEIS2 regulates the expression of Cyclin E/CCNE1 in MM and induction of apoptosis after treatment with the CDK inhibitor Seliciclib/Roscovitine. Interestingly, modulation of MEIS2 can regulate the expression of NKG2D and DNAM-1 NK cell-activating ligands and, importantly, the activity of IMiDs in MM cells. Finally, BETi have the ability to inhibit the expression of MEIS2 in MM, underscoring a novel anticancer activity mediated by these drugs. Our study provides evidence on the role of MEIS2 in MM cell survival and suggests therapeutic strategies targeting of MEIS2 to enhance IMiDs anti-myeloma activity.

## Introduction

MEIS2 is a homeobox transcription factor (TF) member of the Three Amino-acid Loop Extension (TALE) family of homeo-domain-containing transcription factors, important regulators of cell proliferation during development and involved in skeletal muscle differentiation, development of hindbrain and proximal-distal limb patterning^1–4^. Importantly, several evidences demonstrated an oncogenic role for MEIS TFs in the growth and progression of human cancers. Indeed, MEIS1/2 can repress TGF-β type II receptor expression in lung cancer, a major molecular mechanism for inactivation of TGF-β-mediated tumor suppression^5^, and MEIS1/2 can be amplified and overexpressed in ovarian cancers compared with normal ovarian surface epithelium^6,7^. Moreover, MEIS2 affects neuroblastoma proliferation and differentiation, playing a critical role in the control of late cell-cycle genes^8,9^.

On the other hand, tumor expression of MEIS2 confers a more indolent prostate cancer phenotype, with a decreased propensity for metastatic progression, suggesting cancer specific mechanisms^10^.

In leukemia, MEIS2 has been identified as a novel player in Meningioma-1 (MN1)-induced leukemogenesis^11^ and its expression is essential for maintaining myeloid cell lines in an undifferentiated-proliferating state by inhibiting myeloid differentiation^12^.

Little information about the expression, regulation and function(s) of MEIS2 in Multiple Myeloma (MM) is available; however, the expression levels of several members of the HOXA and HOXB gene families together with MEIS1 and MEIS2 have been positively correlated in selected molecular subtypes of MM^13^.

Immunomodulatory drugs (IMiDs) [e.g. Thalidomide, Lenalidomide (Revlimid®) and Pomalidomide (Pomalyst®)] are a class of molecules widely used for treatment of MM. These compounds have direct antitumor effects and act at different levels in MM microenvironment, inducing also remarkable immunomodulatory effects, particularly in T lymphocytes and NK cells^14,15^. The molecular mechanisms mediating these effects remain in part undefined. The cellular target of these drugs is Cereblon (CRBN)^16^, a ubiquitous protein that functions as a substrate receptor for the CUL4-RBX1-DDB1-CRBN E3 ubiquitin ligase (^CRL4^CRBN). IMiDs can alter substrate specificity of CRBN to a number of endogenous cellular targets, redirecting its activity on the recruitment and degradation of novel selected substrates via proteasome, such as IKZF1 and IKZF3, crucial transcription factors (TFs) for MM cell survival^17–19^. In this molecular context, the TF MEIS2 has been identified as an endogenous cellular substrate of CRBN in crystal structure and by biochemical screen^20^. It has been proposed that IMiDs can block CRBN binding to MEIS2 preventing its ubiquitination/degradation, suggesting a role for this protein in modulating IMiD’s anti-MM activity via direct molecular competition. Indeed, strategies able to modify the molecular ratio CRBN/MEIS2 could have a therapeutic relevance and improve anti-MM activity of IMiDs.

Epigenetic modulation is emerging as a promising strategy for cancer therapy^21–23^. Accordingly, small-molecule inhibitors targeting epigenetic modification enzymes can have cytotoxic and differentiation effects on cancer cells^24^. In particular, there is compelling preclinical evidence that small molecule inhibitors of the Bromodomain and Extra-Terminal (BET) proteins, epigenetic readers of acetylated histones (e.g. BRD4), or selective BET-degraders PROTACs (Proteolysis Targeting Chimera) (e.g. ARV-825)^25,26^ can exert antitumor efficacy in refractory hematological malignancies, including MM^27^. Therefore, a number of early-phase, dose-escalation/Phase I trials using different BET-inhibitor compounds covering most hematologic malignancies (including MM) have been completed or are currently underway^28–34^ (https://clinicaltrials.gov/ct2/results?term=bromodomain+inhibitor&Search=Search).

Although BETi are known to regulate the expression of oncogenes and regulators of MM survival (e.g. MYC, IRF4)^28,33^, sensitization to other anti-MM targeted therapies^35–37^ and the recognition by immune effector cells^38^, the characterization of their anticancer molecular targets is still incomplete.

In this work, we investigated different aspects of MEIS2 as a regulator of MM cell biology. Direct repression of MEIS2 by shRNA interference correlated with reduced cell growth, induction of apoptosis and enhanced efficacy of different anti-MM drugs in these cells. Interestingly, inhibition of MEIS2 expression is associated with lower expression of MYC, IRF4 and to a lesser extent IKZF1/3 TFs, important regulators of MM development and progression^17,18,39–41^.

In this context, we characterized the ability of BETi as novel modulators of MEIS2 expression in MM and, in addition, modulation of MEIS2 expression could significantly regulate cell surface levels of the NKG2D and DNAM-1 Natural Killer (NK) cell-activating ligands, and their induction by IMiDs in MM cells.

In summary, these findings underscore novel roles of MEIS2 in MM cell biology and further clarify the molecular mechanisms that regulate direct-antitumor and immuno-mediated activities of BETi and IMiDs against MM.

## Results

### MEIS2 regulates MM cell survival and sensitivity to anti-MM drugs

**Fig. 1:**
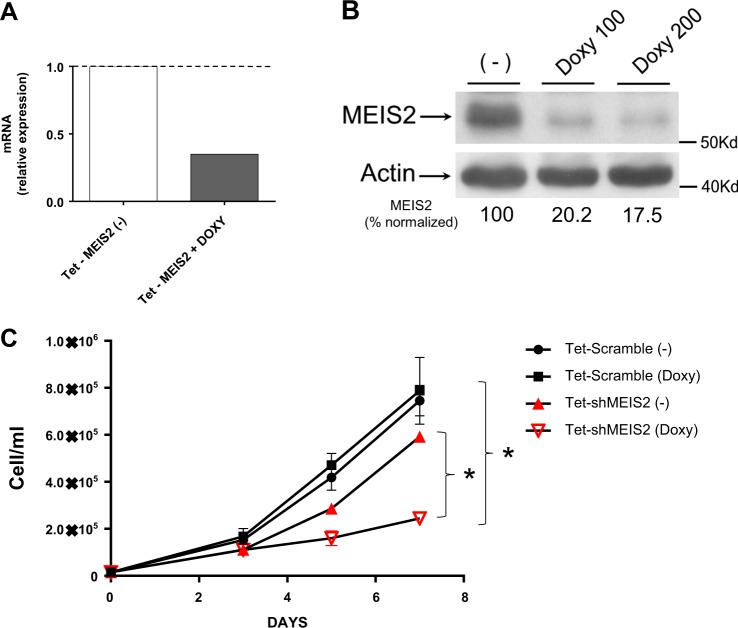
Modulation of MEIS2 expression inhibits survival in SKO-007(J3) human MM cells. **a** Real-Time qRT-PCR analysis of total mRNA obtained from SKO-007(J3)/shMEIS2-Tet cells, untreated or treated with 100 ng/ml Doxycycline for 48 h. Data, expressed as relative mRNA expression, were normalized with GAPDH and referred to the untreated cells considered as calibrator and represent the mean of three experiments. Data are representative of one out of two independent experiments. **b** Lysates of SKO-007(J3)/shMEIS2-Tet cells, untreated or treated with 100 or 200 ng/ml Doxycycline for 48 h, were subjected to western blotting using anti-MEIS2 or anti-βActin antibodies. The proteins transferred to nitrocellulose membranes were stained with Ponceau to verify that similar amounts of proteins had been loaded in each lane. Data are representative of one out of two independent experiments. Densitometric analysis of normalized MEIS2/Actin is shown. **c** Triplicate samples of SKO-007(J3)/shMEIS2-Tet or SKO-007(J3)/sh-Scramble-Tet cells were seeded at 15 × 10^3^/ml, cultured in 12-well plates in the dark, collected and counted after 3, 5 and 7 days. Data are the average of four independent experiments

To investigate the functional significance of MEIS2 expression in MM biology, we silenced MEIS2 expression in the SKO-007(J3) MM human cell line, using lentiviral-transduced small-hairpin RNAs (shRNAs). Using two pre-validated shRNA sequences, we were not able to obtain stable clones with silenced MEIS2 after selection with puromycin, suggesting that basal/sustained expression level of MEIS2 is determinant for MM survival. In order to bypass this limitation, we generated stable transduced SKO-007(J3) cells using a Tet-pLKO-puro-Lentivirus for expression of shRNAs Doxycycline (Doxy)-inducible [SKO-007(J3)/shMEIS2-Tet]. We selected and cloned in Tet-pLKO-puro a highly effective shRNA sequence, able to significantly knockdown MEIS2 expression in the presence of Doxy, as determined by quantitative qRT-PCR (Fig. 1a) and immunoblotting (Fig. 1b). As shown in Fig. 1c, Doxy-induced knockdown of MEIS2 expression in these cells resulted in a significant reduction of cell growth, with no relevant changes in cell cycle and a slight increase of the sub-G1 phase (Supplementary Figure 1A, B). Analysis of microarray public data of MM patients (datasets GSE47552, GSE2113 available at http://www.ncbi.nlm.nih.gov/geo/) did not indicate significant differences of MEIS2 expression among normal PCs, MGUS, Smoldering, MM and plasma cell leukemia (PCL) cells, (shown in Supplementary Fig. 2A, B), suggesting that expression levels of this transcription factor do not correlate with a particular evolving step of these monoclonal gammopathies.

**Fig. 2:**
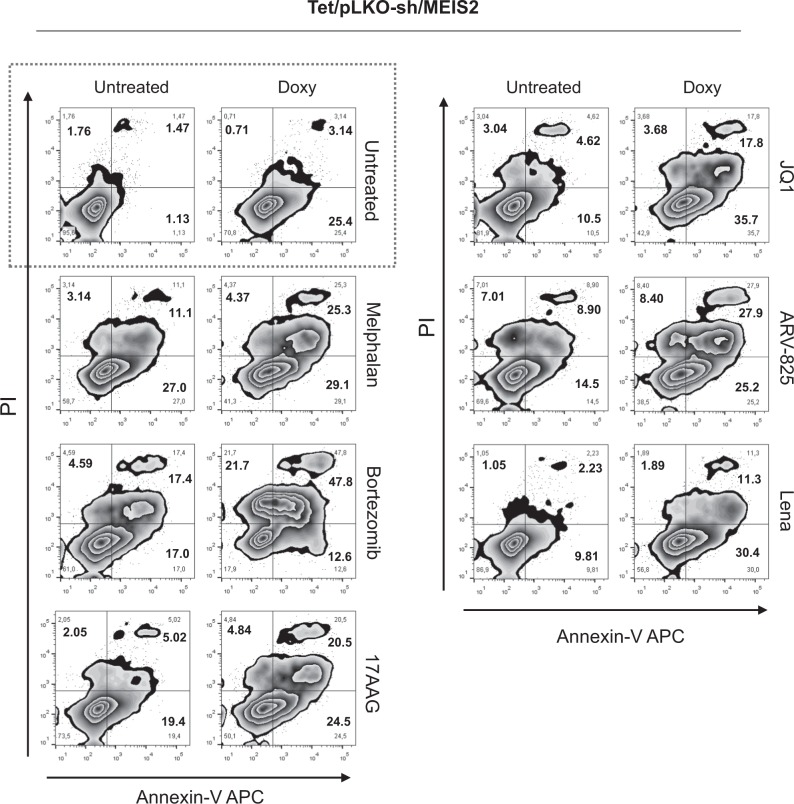
Reduced levels of MEIS2 correlate with increased apoptosis and responsiveness to chemotherapics. Annexin-V assay: SKO-007(J3)/shMEIS2-Tet cells, untreated or treated with 100 ng/ml Doxycycline for 72 h, were incubated with the indicated drug for 24 h (Melphalan 1.5 μM, Bortezomib 2 nM, Lenalidomide 5 μM, 17AAG 0.5 μM, JQ1 0.5 μM, ARV-825 0.2 μM). Basal induction of apoptosis in the presence of Doxy is highlighted in the dotted box. Data are representative of one out of three independent experiments

To explore the possibility that reduction of cell growth by MEIS2 shRNA interference could correlate with increased apoptosis in these cells, or altered regulation of their responsiveness to chemotherapics, we performed Annexin-V assays on SKO-007(J3)/shMEIS2-Tet cells (where expression of MEIS2 was modulated by Doxy) in the presence of selected anti-MM drugs. As shown in Fig. 2, treatment of these cells with Doxy-induced significant apoptosis, and potentiated the effect of several anti-MM drugs able to affect distinct pathways such as genotoxic stress/DDR (Melphalan), proteotoxic stress (Bortezomib), epigenetic regulation (JQ1, ARV-825) or to inhibit/modify the function of specific molecular targets (17AAG/HSP90, Lenalidomide/CRBN). The extent of MEIS2 knockdown-mediated stimulation of apoptosis in response to the different drugs tended to be similar to that seen in the absence of any apoptotic stimuli, suggesting an additive rather than synergistic effect of MEIS2 silencing on drugs-induced cell death.

Interestingly, Gene Ontology enrichment analysis over all the transcripts significantly correlated with MEIS2 (*p*-value < 0.01) in the Hanamura MM Dataset of R2^42^ (http://r2.amc.nl), highlighted several significant gene clusters related to ER homeostasis, gene regulation, apoptosis, mitochondrial functions or senescence, supporting the hypothesis of a role for MEIS2 in different aspects of MM biology (Supplementary Fig. 3).

**Fig. 3:**
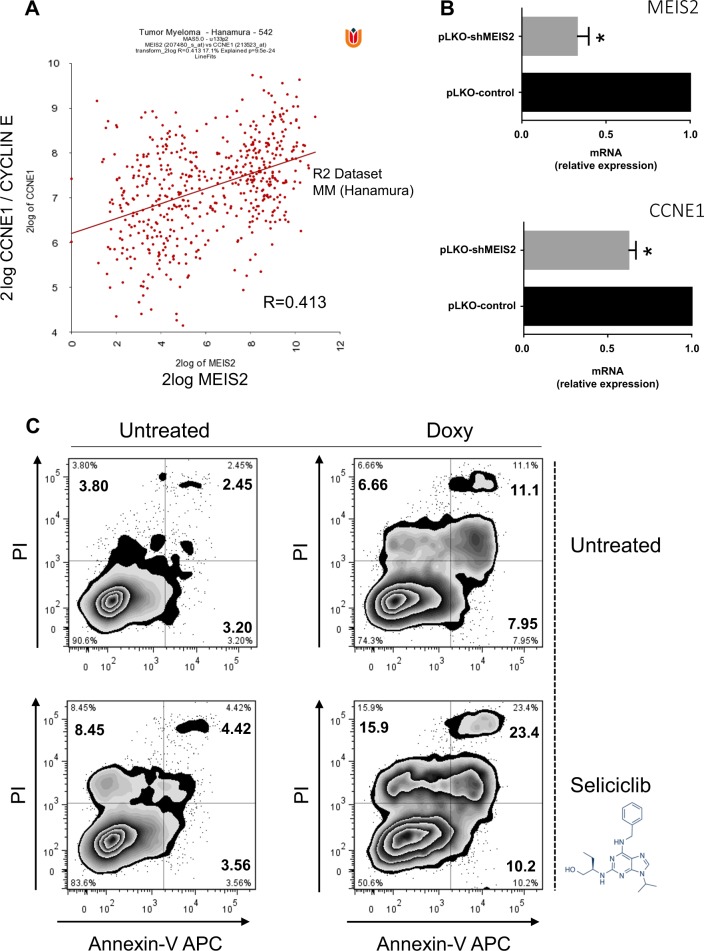
Expression of MEIS2 and correlation with Cyclin E/CCNE1 in MM cells. **a** Correlation analysis of expression values from MM patients (Hanamura MM Dataset of R2), between MEIS2 and CCNE1 (probe set 207480_at for MEIS2 vs. probe set 213523_at for CCNE1). *R*-value: 0.413, *p*-value = 9.5e^−24^. **b** Real-Time qRT-PCR analysis of MEIS2 and CCNE1 on total mRNA obtained from wt-SKO-007(J3) cells transiently transduced with pLKO-shRNA lentiviruses for 48 h. Data, expressed as relative mRNA expression, were normalized with GAPDH and referred to the pLKO-control cells considered as calibrator and represent the mean of three independent experiments (*P < 0.05). **c** Annexin-V assay on SKO-007(J3)/shMEIS2-Tet cells, untreated or treated with 100 ng/ml Doxycycline for 72 h and incubated with Seliciclib/Roscovitine 15 μM for 24 h. Data are representative of one out of three independent experiments

Noteworthy, reduced levels of MEIS2 in Doxy-treated SKO-007(J3)/shMEIS2-Tet cells, increased the production of basal and Melphalan-induced intracellular ROS and the induction of cellular senescence, as evaluated by FACS analysis of H_2_DCFDA and C_12_FDG fluorescence induced by ROS and *β-*Gal activity (Supplementary Figure 4A and B). Moreover, we also found a significant positive correlation between MEIS2 and Cyclin E/CCNE1 expression (R = 0.413 in Hanamura MM Dataset of R2 platform for genomic analysis^42,43^) (Fig. 3a) and confirmed in (Agnelli - R = 0.541 and Chng - R = 0.384 MM Datasets of R2) (Supplementary Figure 5)^42,43^, a regulatory subunit of Cyclin-Dependent Kinase 2 (CDK2). In this regard, MM cells have been shown to be sensitive to CDK inhibition by various chemical inhibitors such as Flavopiridol, SNS-032, P276–00 and Seliciclib/Roscovitine^44^, able to induce cell cycle arrest and apoptosis by downregulation of anti-apoptotic proteins such as MCL1^44^. In particular, a pro-apoptotic response of MM cells to Seliciclib/Roscovitine inversely correlates to Cyclin E/CCNE1 expression levels^44^. In agreement with the positive correlation observed in the Hanamura MM Dataset, our data indicate that reduced levels of MEIS2 in wt-SKO-007(J3) cells obtained by transient transduction of validated shRNA sequences, significantly repressed mRNA expression of Cyclin E/CCNE1 in these cells (Fig. 3b) and, importantly, increased the induction of apoptosis in the presence of Seliciclib/Roscovitine (Fig. 3c), suggesting that combined inhibition of specific CDKs and targeting of MEIS2 could represent an interesting therapeutic strategy in MM.

Collectively, these data indicate that basal expression of MEIS2 is critical for MM survival and resistance to anti-MM chemotherapics.

### MEIS2 is an endogenous regulator of IMiDs activity in MM

IMiDs binding to CRBN alter the function of the ubiquitin ligase ^CRBN^CRL4; in particular, enforced binding of IMiDs to CRBN is able to disrupt the recruitment of endogenous substrates (e.g. MEIS2) and, at the same time, promotes the ubiquitination of other proteins by formation of a novel neomorphic structure with different specificity and affinity^20,45^. In this context, IMiDs can block CRBN binding to MEIS2 preventing its ubiquitination and degradation^20^. This mechanism suggests a possible role for MEIS2 in modulating IMiDs anti-MM activity. We reasoned that differential modulation of MEIS2 expression in MM cells could be an important determinant for CRBN/IMiDs activity. In this regard, our laboratory has recently characterized the capability of IMiDs to upregulate cell surface expression of the NKG2D and DNAM-1 NK cell-activating ligands MICA and PVR/CD155 on MM cells, thus enhancing NK cell recognition and killing. This pathway is mediated by inhibition of IKZF1/3 and IRF4 expression, “druggable” transcriptional repressors of these genes^46,47^.

**Fig. 4:**
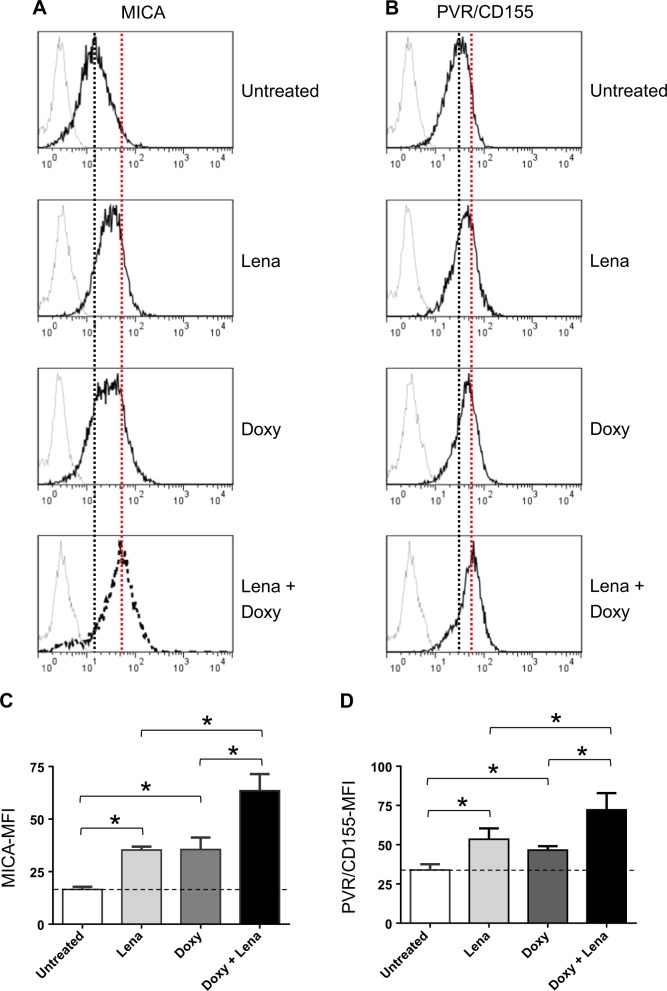
MEIS2 regulates MICA and PVR/CD155 cell surface expression and their upregulation by IMiDs in MM cells. **a** MICA and **b** PVR/CD155 cell surface expression were analyzed by flow cytometry on SKO-007(J3)/shMEIS2-Tet cells, untreated or treated with 100 ng/ml Doxycycline, 5 μM Lenalidomide or the combination of the two for 72 h. Data are representative of one out of three independent experiments. **c**, **d** Histograms represent MFI of specific mAb - MFI of isotype control. The MFI of MICA and PVR/CD155 were calculated based on at least three independent experiments and evaluated by paired Student’s *t* test (*P < 0.05)

In order to investigate the possible role of MEIS2 as a functional molecular competitor/regulator of IMiDs in our model, we studied SKO-007(J3)/shMEIS2-Tet cells in the presence of Lenalidomide + Doxy. We found that reduced cellular levels of MEIS2 in Doxy-treated cells increased basal expression of the NKG2DL MICA and, to a lesser extent, of the DNAM-1 ligand PVR/CD155. Moreover, low expression of MEIS2 potentiated the activity of Lenalidomide on the expression of these ligands, as shown in the Fig. 4a–d.

**Fig. 5:**
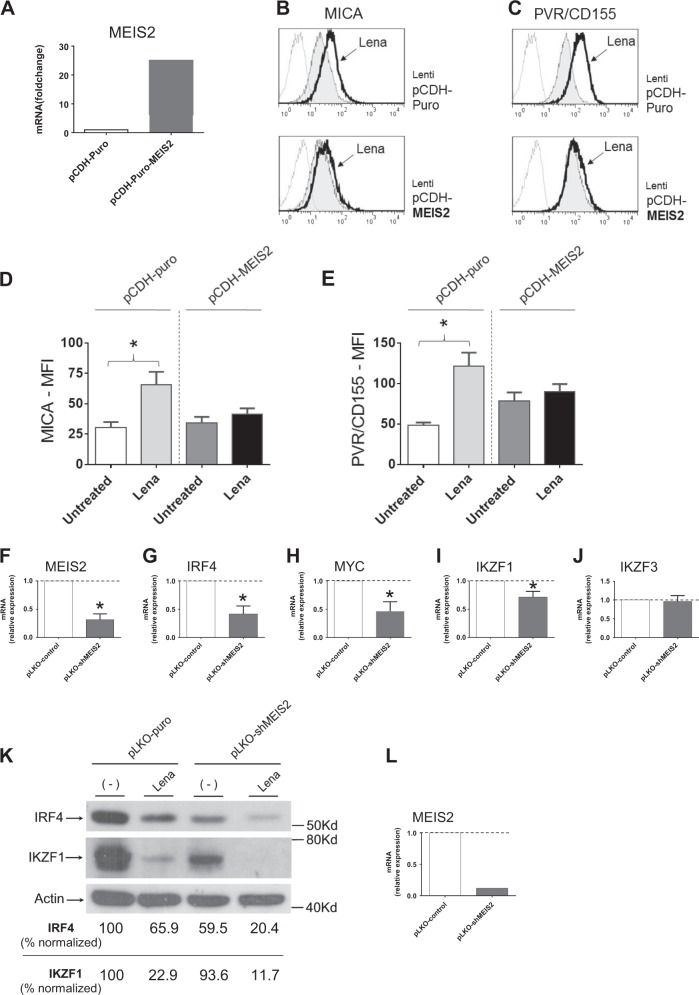
MEIS2 inhibits activity of IMiDs in MM. **a** Real-Time qRT-PCR analysis for MEIS2 on total mRNA obtained from wt-SKO-007(J3) cells transiently infected with the lentiviral vector pCDH-puro-Flag-MEIS2d or the control empty vector pCDH-CMV-puro for 72 h. Data, expressed as relative mRNA expression, were normalized with GAPDH and referred to the pCDH-CMV-puro infected cells considered as calibrator. A representative experiment is shown in the figure. **b** MICA and (**c**) PVR/CD155 cell surface expression were analyzed by flow cytometry on wt-SKO-007(J3) cells, transiently infected with the lentiviral vectors pCDH-puro-Flag-MEIS2d or the control empty vector pCDH-CMV-puro, and treated with 5 μM Lenalidomide for 72 h. Data are representative of one out of three independent experiments. **d**, **e** Histograms represent MFI of specific mAb - MFI of isotype control. The MFI of MICA and PVR/CD155 were calculated based on at least three independent experiments and evaluated by paired Student’s *t* test (*P < 0.05). **f**–**j** MEIS2 regulates IRF4, MYC and IKZF1 mRNA expression in MM. Total RNA was isolated from transiently infected wt-SKO-007(J3) cells as indicated in the figure and analyzed by Real-Time qRT-PCR. Data, expressed as relative mRNA expression, were normalized with GAPDH and referred to the cells infected with non-target shRNA (pLKO-control) considered as calibrator and represent the mean of three independent experiments (*P < 0.05). **k** Lysates of wt-SKO-007(J3) cells transiently infected as indicated in the figure for 48 h were untreated or treated with Lenalidomide (48 h) and subjected to western blotting using anti-IRF4, anti-IKZF1 or anti-Actin antibodies. In all experiments, proteins transferred to nitrocellulose membranes were stained with Ponceau to verify that similar amounts of proteins had been loaded in each lane. Data are representative of one out of two independent experiments. Densitometric analysis of normalized IRF4/Actin and IKZF1/Actin is shown. **l**) Total RNA was isolated from a fraction of the transiently infected wt-SKO-007(J3) cells used in panel (K) and analyzed by Real-Time qRT-PCR for MEIS2 expression

On the contrary, lentiviral-mediated transient overexpression of MEIS2 in SKO-007(J3) cells abrogated upregulation of MICA and PVR/CD155 in Lenalidomide treated cells (Fig. 5a–e), in agreement with the hypothesis of MEIS2 as a functional intracellular competitor of IMiDs activity in MM.

Interestingly, knockdown of MEIS2 inhibited mRNAs expression of IRF4, MYC and to a lesser extent IKZF1 in these cells (Fig. 5f–j), underscoring a possible direct regulatory role of MEIS2 on the expression of these transcription factors. In addition, as shown in Fig. 5k, transient knockdown of MEIS2 potentiated the repressive effect on IRF4 and IKZF1 expression mediated by Lenalidomide in the same experimental setting.

These data could explain, at the same time, the two main findings described above: increased apoptosis and lower resistance to anti-MM drugs (protective role mediated by IRF4, MYC, IKZF1/3), and the higher expression of NK cell-activating ligands in the presence of IMiDs (repressive role of IRF4, IKZF1 on NK cell-activating ligands), caused by reduced MEIS2 expression in these cells.

### BETi modulate expression of MEIS2 in human MM cells

**Fig. 6:**
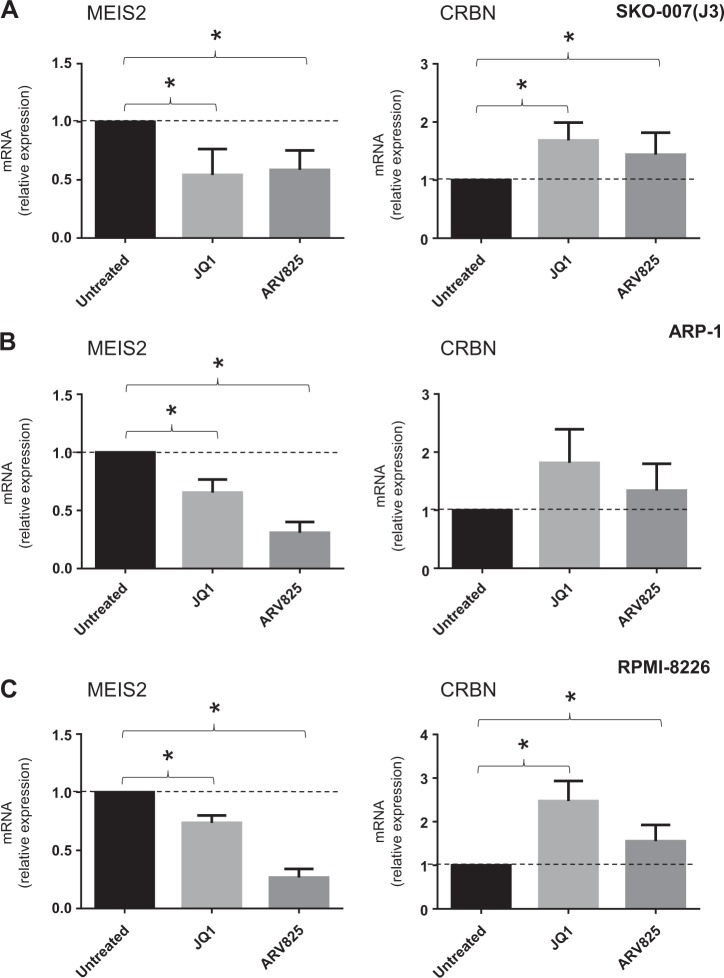
JQ1/BETi and PROTAC/BRD4 degrader ARV-825 modulate MEIS2 mRNA expression in MM cells. **a**–**c** Real-Time qRT-PCR analysis of MEIS2 and CRBN on total mRNAs obtained from SKO-007(J3), ARP-1 and RPMI-8226 cells, untreated or treated with JQ1 (0.5 μM) or ARV-825 (0.2 μM) for 48 h. Data, expressed as relative mRNA expression, were normalized with GAPDH and referred to the untreated cells considered as calibrator and represent the mean of three experiments (*P < 0.05)

**Fig. 7:**
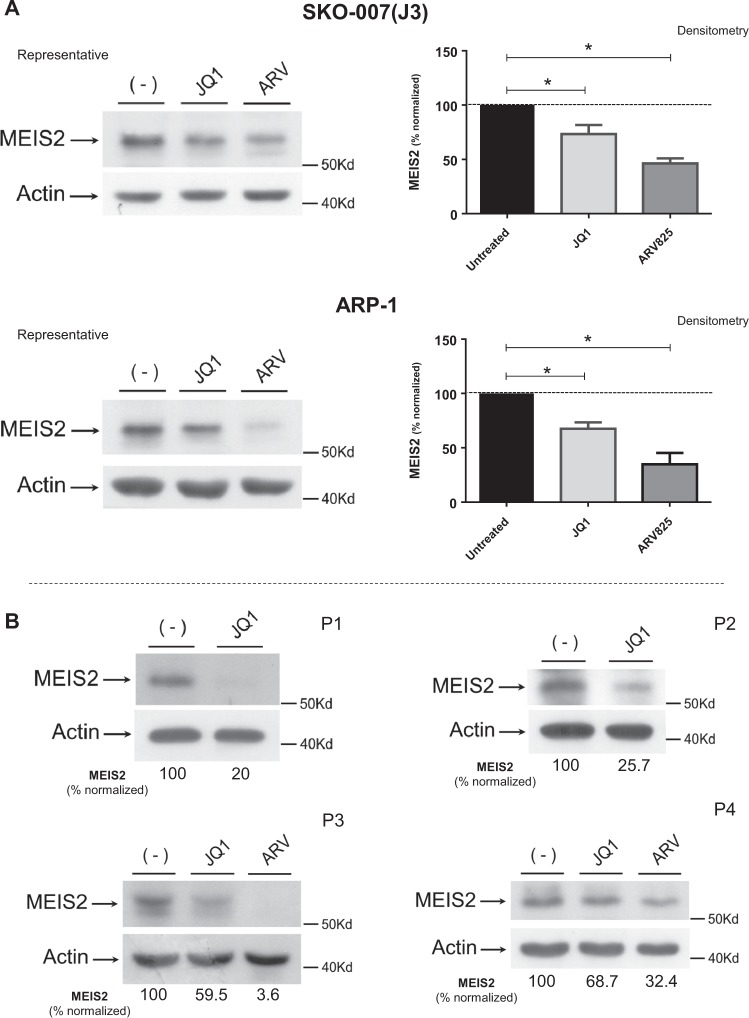
JQ1/BETi and PROTAC/BRD4 degrader ARV-825 modulate MEIS2 protein expression in MM cell lines and malignant PCs from MM patients. **a** SKO-007(J3) or ARP-1 cell cells untreated or treated with JQ1 (0.5 µM) or ARV-825 (0.2 µM) for 48 h, were subjected to western blotting using anti-MEIS2 or anti-Actin antibodies. Left: WB data representative of one out of three independent experiments. Right: densitometric analysis of normalized MEIS2/Actin of the different experiments (*P < 0.05). **b** Lysates of MM cells purified from four patients, untreated or treated as described above, were subjected to western blotting using anti-MEIS2 or anti-Actin antibodies. Densitometric analysis of normalized MEIS2/Actin is shown

Based on the results shown above and given the possible clinical relevance of strategies able to modify the molecular ratio CRBN/MEIS2 and improve anti-MM activity of IMiDs, we investigated the ability of BETi to regulate the expression of MEIS2 in MM cells. Different human MM cell lines [SKO-007(J3), ARP-1 and RPMI-8226] were treated with JQ1, a small molecule bromodomain inhibitor displaying potent binding affinity to BET family proteins^48^, and with ARV-825, a PROTAC consisting of a BRD4 binding moiety (triazolo-diazepine acetamide class in the BETi OTX015) chemically linked to the IMiD Pomalidomide, a structure able to recruit BRD4 to CRBN and to induce its rapid degradation via the proteasome^25,26^. As shown in Fig. 6a–c, treatment of MM cell lines with these drugs inhibited basal mRNA expression of MEIS2 and, interestingly, with apparent no inhibitory effect on the expression of CRBN. Inhibition of MEIS2 was also confirmed in SKO-007(J3) and ARP-1 cells by western-blot analysis (Fig. 7a) and, importantly, in malignant plasma cells isolated from MM patients (Fig. 7b). Noteworthy, analysis of microarray public data (datasets GSE44929 and GSE31365 available at http://www.ncbi.nlm.nih.gov/geo/) showed relevant changes of MEIS2 mRNA expression in human MM cell lines (MM1.S and KMS11) treated with JQ1 (micromolar range) (Supplementary Figure 6A–C), in agreement with our experimental observations. Moreover, a significant downregulation of the BRD4 signal on MEIS2 TSS region was revealed in the presence of JQ1, by CHIP-Seq analysis of available datasets (GSE44931/GSE42355) obtained in the MM1.S cell line^28^ (Supplementary Figure 7).

Altogether, these data indicate that BETi or selective degradation of BRD4 by ARV-825/PROTAC can modulate MEIS2 mRNA and protein expression in human MM cells.

### Expression of MEIS2 regulates degradation of CRBN^Lena^ targets in MM cells

**Fig. 8:**
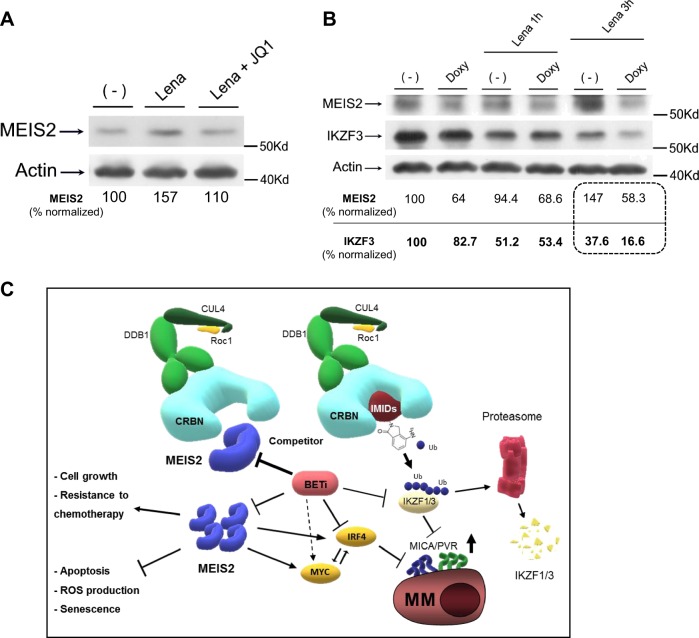
Expression of MEIS2 regulates degradation of CRBN^Lena^ targets in MM cells. **a** Lysates of wt-SKO-007(J3) cells untreated or treated with 5 μM Lenalidomide, 0.5 μM JQ1 or the combination of the two for 48 h, were subjected to western blotting using anti-MEIS2 or anti-Actin antibodies. Data are representative of one out of two independent experiments. Densitometric analysis of normalized MEIS2/Actin is shown. **b** Lysates of SKO-007(J3)/shMEIS2-Tet cells, untreated or treated with 100 ng/ml Doxycycline for 72 h and stimulated with 5 μM Lenalidomide for 1 h and 3 h were subjected to western blotting using anti-MEIS2, anti-IKZF3 or anti-Actin antibodies. Densitometric analysis of normalized MEIS2/Actin and IKZF3/Actin is shown. Data are representative of one out of two independent experiments. **c** Proposed model for MEIS2-IMiDs-BETi interplay in MM cells: repression of MEIS2 correlates with reduced cell growth, induction of apoptosis and enhanced efficacy of different anti-MM drugs. Moreover, modulation of MEIS2 enhances the activity of IMiDs and increases expression of NK cell-activating ligands in MM cells. These findings underscore novel roles of MEIS2 in MM cell biology and further clarify the molecular mechanisms that regulate direct-antitumor and immuno-mediated activities of BETi and IMiDs against MM

We had previously shown that the combined treatment BETi + IMiDs can further repress mRNA expression of IRF4 and enhance the expression of the NKG2DL MICA in MM, suggesting that CRBN-mediated degradation of IKZF1/3 cooperates with BETi mediated repression of IRF4/MYC in this pathway^38^. We confirmed the efficacy of the combination of JQ1 + Lenalidomide and, as shown in Supplementary Figure 8, this treatment further increased apoptosis in different MM cell lines and reduced levels of MYC mRNA expression in the same experimental setting (Supplementary Figure. 9). Interestingly, treatment of SKO-007(J3) cells with Lenalidomide upregulated the expression levels of MEIS2, in agreement with the observation of Fischer E.S. et al.^20^ on the degradation of MEIS2 via CRBN, and the presence of JQ1 significantly abrogated this upregulation (Fig. 8a). These data suggest that this mechanism could cooperate with the already described repressive activity of these drugs on IRF4 and IKZF1/3 expression, reducing the amount of the endogenous competitor MEIS2 bound to CRBN and potentiating its selective anti-MM CRL4-ubiquitin ligase activity. Accordingly, in a different experimental setting, western-blot analysis revealed that reduced levels of MEIS2 in Doxy-treated SKO-007(J3)/shMEIS2-Tet cells could further enhance degradation of IKZF3 by Lenalidomide, a canonical biomarker of CRBN^Lena^ activity in MM (Fig. 8b).

These observations suggest that specific strategies or drugs able to modulate either MEIS2 expression or its ability to bind CRBN could improve the activity of IMiDs in MM patients.

## Discussion

In this study, we investigated on the role of the transcription factor MEIS2 in MM, the activity of BETi or PROTAC-mediated Inhibition/degradation of BET proteins on its expression, and the functional implications of its modulation on the activity of selected chemotherapics, focusing on IMiDs.

The data shown in this manuscript indicate that reduced expression of MEIS2 can significantly inhibit proliferation and induce cell death, with no relevant effects on the cell cycle. Interestingly, a similar modality of cell death was previously described in MM cells after IRF4 knockdown, a critical transcription factor regulator, together MYC, of many genes involved in cell survival/apoptosis in MM^40^ and downregulated by MEIS2 shRNA interference (Fig. 5f–h). Reduced expression of MEIS2 also increased the activity of different anti-MM chemotherapics able to affect molecular pathways critical for MM biology such as genotoxic and proteotoxic stress (e.g. Melphalan, Bortezomib), the function of molecular chaperones important for correct folding and function of client proteins (e.g. 17AAG/HSP90), the activity of epigenetic readers of acetylated histones (e.g. JQ1/BRD4), or to modify the function of molecular targets (e.g. Lenalidomide/CRBN) (Fig. 2).

In the context of genotoxic stress and activation of DDR, we observed that modulation of MEIS2 expression correlated with increased production of ROS and increased cellular senescence, in the presence of Melphalan. In this regard, Gene Ontology enrichment analysis over transcripts significantly correlated with MEIS2 [MM dataset of R2 (Hanamura)]^42^, highlighted several gene clusters related to apoptosis, ER homeostasis, mitochondrial functions and senescence, supporting the hypothesis of multiple regulatory roles mediated by MEIS2 in critical aspects of MM biology (Supplementary Figure 3). Noteworthy, we also found a significant positive correlation between MEIS2 and Cyclin E/CCNE1 expression (Fig. 3a and Supplementary Figure 5) and, reduced levels of MEIS2 by lentiviral-mediated shRNA interference increased apoptosis in the presence of Seliciclib/Roscovitine (Fig. 3b, c), suggesting that selective CDK inhibition combined with MEIS2 targeting could represent an interesting novel therapeutic strategy. Further experiments will be needed to investigate the possible cooperation with the cyclin-dependent kinase (CDK) 4/6-specific inhibitor Palbociclib (PD-0332991), able to enhance the cytotoxic effect of different drugs effective in MM, including Bortezomib and Lenalidomide, as confirmed in early-phase clinical trials (reviewed in^49,50^).

No data are available regarding the possibility to modulate the expression of MEIS2 in MM.

Based on the antiproliferative and pro-apoptotic effects of reduced MEIS2 levels, and the BETi-mediated anticancer activities against MM^33,51^, we speculated that the epigenetic readers of acetylated histones, BET proteins, could regulate the expression of MEIS2 in MM. Indeed, treatment with JQ1 significantly inhibited expression of MEIS2 in different human MM cell lines, both at the mRNA and protein levels (Figs. 6 and 7a) and in neoplastic plasma cells isolated from different MM patients (Fig. 7b). This observation was also confirmed by analysis of microarray public data of MM patients (datasets GSE44929 and GSE31365 available at http://www.ncbi.nlm.nih.gov/geo/), showing relevant modulation of MEIS2 expression in human MM cell lines treated with JQ1 (Supplementary Figure 6). These data indicate that BETi or selective degradation of BRD4 by specific PROTACs can modulate MEIS2 expression in human MM cells and suggest a possible cross-talk between MEIS2 and BET proteins in MM, where the repression of this transcription factor by this class of epigenetic drugs can enhance anticancer activity increasing apoptosis.

A regulatory network connecting MEIS2 and immunomodulation by IMiDs in MM, has been also described in this work. We had already shown in a previous paper that IMiDs have the ability to upregulate the expression of NK cell-activating ligands in MM cells, a pathway involving a mechanism of derepression of MICA and PVR/CD155 genes by ^IMiD^CRBN-mediated degradation of IKZF1/3 and inhibition of IRF4 expression^46^. Using this experimental model, we found that reduced cellular levels of MEIS2 (in Doxy-treated SKO-007(J3)/shMEIS2-Tet) increased basal expression of the NKG2DL MICA and, to a lesser extent, of the DNAM-1 ligand PVR/CD155. Knockdown of MEIS2 inhibited mRNA expression of IRF4, MYC and to a lesser extent IKZF1/3 (Fig. 5f–j), inducing in this way a mechanism of derepression of these ligands as reported for Lenalidomide^46^. Accordingly, lower expression of MEIS2 potentiated Lenalidomide-induced upregulation of these ligands, as shown in Fig. 4.

These data can explain the observed increase of MICA and PVR/CD155 expression and, at the same time, the antiproliferative/pro-apoptotic activity of reduced expression of MEIS2 in combination with different chemotherapics, both mediated by downregulation of IRF4 and MYC, important regulators of transcriptional networks involved in MM survival, drug-resistance and progression^17,18,39–41^.

An additional interplay between MEIS2, IMiDs and BET proteins in this model concerns the possibility to modulate the molecular competition described for MEIS2 and IMiDs on the activity of CRBN^20^. As shown in Fig. 8a, the presence of JQ1 could block the upregulation of MEIS2 induced by Lenalidomide^20^ and, lower levels of MEIS2 correlated with increased degradation of CRBN^Len^ targets as shown for IKZF3 (Fig. 8b). On the contrary, overexpression of MEIS2 inhibited the activity of Lenalidomide as observed for the upregulation of MICA and PVR/CD155 in treated cells (Fig. 5a–e).

This molecular pathway could be important in the context of the different activities mediated by IMiDs on key aspects of MM cell biology. It could enhance their direct anti-MM activity, increase the expression of NK cell-activating ligands on MM cells and, importantly, improve the action mediated by IMiDs on NK-cell effector functions. Indeed, enhanced activity of IMiDs could lower the threshold concentrations of ligands expressed on tumor cells needed for optimal activation of several activating receptors (e.g. CD16 or NKG2D), by increasing nanoscale rearrangements in cortical actin at the NK-target cell immunologic synapse, as already described in^52^. Further experiments will be needed to investigate and confirm this immunomodulatory pathway in the context of NK cell-mediated anti-MM immune response.

The results shown in this work underscore novel roles of MEIS2 in MM biology, as a regulator of cell growth and resistance to apoptosis, and describe a molecular pathway where its modulation could increase tumor recognition and killing by the immune system.

The novel finding that BET inhibitors (or BRD4 degraders) have the ability to repress the expression of this gene, together with the observation that reduced expression of MEIS2 can increase selected activities of IMiDs, further extends the current knowledge on the anticancer potential of these drugs and suggest that strategies aimed to target the expression/function of this protein could improve therapeutical approaches in MM.

## Materials and methods

### Cell lines and clinical samples

Human myeloma cell lines SKO-007(J3), ARP-1, RPMI-8226, MM1.S, U266 and JJN3 have been already described^46,53^ and were kindly provided by Prof. P. Trivedi (University of Rome, Sapienza, Italy) and Prof. N. Giuliani (University of Parma, Italy). After thawing, cells were cultured at 37 °C and 5% CO2 in RPMI 1640 supplemented with 10% FCS, 2 mM L-glutamine, 100 U/ml penicillin and 100 U/ml streptomycin (complete medium) for no longer than 4 weeks and tested for mycoplasma monthly (EZ-PCR Mycoplasma Test Kit, Biological Industries - Kibbutz Beit Haemek, Israel). These cell lines were authenticated by IRCCS Azienda Ospedaliera Universitaria San Martino-IST, S.S. Banca Biologica e Cell factory (Genova, IT). Cells with stable knockdown of MEIS2 were created by lentiviral-mediated transduction of SKO-007(J3) cells with Tet/pLKO-shMEIS2 or Tet-pLKO-puro-Scrambled vectors. After selection with Puromycin (0.5 to 1 µg/ml), stable bulk selected cells were maintained at 37 °C and 5% CO_2_ in RPMI 1640 supplemented with 10% fetal bovine serum (FBS) approved for use with Tet-inducible systems, 2 mM L-glutamine, 100 U/ml penicillin and 100 U/ml streptomycin (complete medium). To trigger shRNA-MEIS2 expression we added Doxycycline hydrate (100 ng/ml) to the growth medium for the indicated time. SKO-007(J3)/shMEIS2-Tet cells and SKO-007(J3)/sh-Scramble-Tet cells were maintained as described above and with 10% foetal bovine serum (FBS) approved for use with Tet-inducible systems. The human 293 T embryonic kidney cells were purchased from ATCC and were maintained in Dulbecco’s modified Eagle’s supplemented with 10% FCS.

Bone marrow samples from patients with MM were managed at the Division of Hematology, Department of Cellular Biotechnologies and Hematology, University of Rome, Sapienza, Italy (Supplementary Figure 10). Informed consent in accordance with the Declaration of Helsinki was obtained from all patients, and approval was obtained from the Ethics Committee of the Sapienza University of Rome (Rif. 3373). The bone marrow aspirates were processed as already described in^46^. In some experiments, myeloma cells were selected using anti-CD138 magnetic beads (Miltenyi Biotec. S.r.l. Bologna, IT) More than 95% of the purified cells expressed CD138 and CD38.

### Reagents and Antibodies

The bromodomain inhibitor JQ1 and Bortezomib (PS-341) were purchased from Selleckchem.Com (Munich, Germany). Lenalidomide was purchased from BioVision Inc. (Milpitas, California USA). The hetero-bifunctional PROTAC (Proteolysis Targeting Chimera) ARV-825, was purchased from Chemieteck (Indianapolis, IN, USA). These drugs were dissolved in dimethylsulphoxide (DMSO) and stored at −20 °C until use. The final concentration of DMSO in all experiments was < 0.1%. Roscovitine a potent and selective inhibitor of cyclin-dependent kinases and the antibiotic Doxycycline hydrate (used to induce SKO-007(J3)/shMEIS2-Tet cells), Melphalan and 17-AAG (17-allylaminogeldanamycin) were purchased from Sigma-Aldrich S.r.l. (Milan, IT). The following monoclonal antibodies (mAbs) were used for immunostaining or as blocking Abs: anti-MICA (MAB159227) was purchased from R&D System (Minneapolis, MN), anti-PVR/CD155 (SKII.4) kindly provided by Prof. M. Colonna (Washington University, St Louis, MO), APC Goat anti-mouse IgG (Poly4053), was purchased from BioLegend (San Diego, CA). Anti-CD138-FITC (M15) and anti-CD38-APC (HIT2) were purchased from BD Biosciences (San Jose, CA).

### Flow cytometry

SKO-007(J3) cells or SKO-007(J3)/shMEIS2-Tet cells were cultured in 6-well tissue culture plates at a concentration of 2 × 10^5^ cells/ml in the presence of the indicated drug(s). The expression of the NKG2D and DNAM-1 ligands on MM cells was analyzed by immunofluorescence staining using unconjugated mAbs, followed by secondary GAM-APC. In all experiments, cells were stained with Propidium Iodide (PI) (1 µg/ml) in order to assess cell viability (always higher than 90% after the different treatments). Nonspecific fluorescence was assessed by using an isotype-matched irrelevant mAb R&D System, followed by the same secondary antibody. Fluorescence was analyzed using a FACSCalibur flow cytometer BD Biosciences and data were analyzed using FlowJo Flow Cytometric Data Analysis Software (FlowJo - Ashland, OR). The analysis of ligand expression on patient derived plasma cells was performed by gating on the CD138^+^ and CD38^+^ PC population.

### Plasmids

For knocking down MEIS2 we used the following lentiviral vectors/sequences: pLKO.1-sh-MEIS2 (TRCN0000274058), (TRCN0000016044), and the control vector pLKO non-targeting shRNA (MISSION™ Sigma-Aldrich). The inducible lentiviral vectors Tet-pLKO-puro and Tet-pLKO-puro-Scrambled (containing all the necessary components for the inducible expression of shRNA in target cells) were a gift from Dr. Dmitri Wiederschain (Addgene plasmid #21915) and Dr. Charles Rudin (Addgene plasmid #47541). To generate inducible Tet-pLKO-puro/shMEIS2 vector we cloned the MEIS2 - TRCN0000274058 sequence (from MISSION™ Sigma-Aldrich) into pLKO-Tet-On vector (EcoRI-AgeI). The lentiviral expression vector pCDH-puro-Flag-MEIS2d and the control vector pCDH-CMV-puro were kindly provided by Dr. H.F. Ding (Georgia Cancer Center - Georgia, USA).

### DNA transfections, virus production and in vitro transduction

For lentivirus production, lentiviral vectors were cotransfected together the packaging vectors pVSVG and psPAX2 into 293 T cells using Lipofectamine Plus (Life Technologies Italia – Milan, IT). After transfection, cells were placed in fresh medium. After a further 48-hour culture, virus-containing supernatants were harvested, filtered and used immediately for infections. Infections were performed on 0.5 × 10^6^ SKO-007(J3) cells in 2 ml complete medium with Polybrene (8 μg/ml) (Hexadimethrine bromide - Sigma-Aldrich) for 2 h.

### mRNA detection and quantitative Real-Time polymerase chain reaction (qRT-PCR)

Total RNA was extracted using TRIZOL™ Life Technologies, according to manufacturer’s instructions. The concentration and quality of the extracted total RNA was determined by measuring light absorbance at 260 nm (A260) and the ratio of A260/A280. Reverse transcription was carried out in a 25 µl reaction volume with 2 µg of total RNA according to the manufacturer’s protocol for M-MLV reverse transcriptase (Promega – Promega Italia, Milan, IT). cDNAs were amplified (TaqMan assays) in triplicate with primers for MICA (Hs00792195_m1), IRF4 (Hs01056533_m1), MYC (Hs00153408_m1), IKZF1 (Hs00958474_m1), IKZF3 (Hs00232635_m1), MEIS2 (Hs 00542638_m1), CRBN (Hs00372271_m1), CCNE1 (Hs01026536_m1) and GAPDH (Hs03929097_g1) conjugated with fluorochrome FAM (Applied Biosystems Italia – Milan, IT). The level of expression was measured using Ct (threshold cycle). The Ct was obtained by subtracting the Ct value of the gene of interest from the housekeeping gene (GAPDH) Ct value. In the present study, we used Ct of the untreated sample as the calibrator. The fold change was calculated according to the formula 2^−ΔΔCt^, where ΔΔCt was the difference between Ct of the sample and the Ct of the calibrator (according to the formula, the value of the calibrator in each run is 1). The analysis was performed using the SDS version 2.4 software (Applied Biosystems). All PCR reactions were performed using a StepOnePlus™ Real-Time PCR System (Applied Biosystems).

### Apoptosis and cell cycle analysis

Apoptotic cell death was evaluated using APC Annexin-V Apoptosis Detection Kit with PI (BioLegend). Briefly, SKO-007(J3)/shMEIS2-Tet cells seeded 3 × 10^4^/ml were cultured in 12-well plates, untreated or treated with 100 ng/ml Doxycycline for 72 h, and then incubated with different drugs or vehicle for 24 h. Cells were then stained using Annexin-V/APC and PI according to the manufacturer’s instruction. Cell populations were acquired using FACSCantoII flow cytometer (BD Biosciences). Flow cytometric analysis was performed using FlowJo Flow Cytometric Analysis Software. To evaluate the cell cycle, cells were harvested, washed in PBS with 0.1% sodium azide, fixed in cold 70% ethanol and incubated at −20 °C o/n. Thereafter, in order to remove ethanol and precipitated protein, cells were washed twice with PBS. Cells were incubated with a solution containing RNAse A (1 mg/mL) (Sigma-Aldrich S.r.l. - Milan, IT) for 1 h and then with propidium iodide (50 μg/mL) for 30 min at R/T. Cells were acquired using FACSCantoII flow cytometer (BD Biosciences). Flow cytometric analysis was performed using FlowJo Flow Cytometric Analysis Software.

### Analysis of cellular senescence

Senescence-associated β-galactosidase assay was performed using the fluorogenic substrate 5-dodecanoylaminofluorescein di-β-D-galactopyranoside (C_12_FDG) (Invitrogen, Frederick, MD) to measure β-galactosidase activity by flow cytometry. Cells were incubated 1 h with 100 nM Bafilomycin A1 to induce lysosomal alkalinization, followed by 1 h incubation with C_12_FDG (33 μM). The C_12_-fluorescein signal of senescent cells was measured on the FL-1 detector using a FACSCantoII flow cytometer (BD Biosciences). Flow cytometric analysis was performed using FlowJo Flow Cytometric Analysis Software.

### Reactive oxygen species (ROS) production

To evaluate ROS levels, treated cells were stained with H_2_DCF-DA (Sigma-Aldrich), a non-fluorescent compound under normal conditions that becomes fluorescent when oxidized by ROS, according to standard protocols. Cells were rinsed twice with PBS and incubated with H_2_DCFDA (10 μM) for 30 min at 37 °C. Cell fluorescence was detected in FL1 channel and analyzed with a FACSCanto II (BD Biosciences). Data were analyzed using FlowJo Flow Cytometric Analysis Software.

### Proliferation assay

To evaluate the arrest of cell growth after silencing of shMEIS2, triplicate samples of SKO-007(J3)/shMEIS2-Tet and the control Tet-Scramble cells seeded as (15 × 10^3^/ml) were cultured in 12-well plates (in the dark) in the presence or in the absence of 100 ng/ml Doxycycline, collected and counted at different days (3, 5 and 7 days) by trypan-blue cell counting.

### Western blot analysis

For Western-Blot analysis, SKO-007(J3) cells or patient derived myeloma cells (isolated using anti-CD138 magnetic beads Miltenyi Biotec) were pelleted, washed once with cold phosphate-buffered saline, resuspended in lysis buffer [1% Nonidet P-40 (v/v), 10% glycerol, 0.1% SDS, 0.5% Sodium Deoxycholate, 1 mM phenyl-methyl-sulfonyl fluoride (PMSF), 10 mM NaF, 1 mM Na_3_VO_4_, complete protease inhibitor mixture Roche in PBS] and subsequently incubated 30 min on ice. The lysate was centrifuged at 14000 g for 15 min at 4 °C and the supernatant was collected as whole cell extract. Protein concentration was determined by the BCA method (Pierce – ThermoFisher Scientific, Milan, IT). Thirty to 50 μg of cell extract was run on 10% denaturing SDS-polyacrylamide gels. Proteins were then electroblotted onto nitrocellulose membranes Schleicher&Schuell (Dassel, Germany), stained with Ponceau to verify that similar amounts of proteins had been loaded in each lane, and blocked in 5% BSA in TBST buffer. Immunoreactive bands were visualized on the nitrocellulose membranes, using horseradish-peroxidase-coupled goat anti-rabbit or goat anti-mouse immunoglobulins and the ECL Prime western blotting System (Sigma Aldrich), following the manufacturer’s instructions. Antibodies against *β*-actin, IRF4 (H-140), IKZF1 (H-100), IKZF3 (L-15) and MEIS2 (63-T) were purchased from Santa Cruz Biotechnology (Heidelberg, Germany). Antibody against CRBN (HPA045910) was purchased from Sigma-Aldrich.

### Statistical analysis

Error bars represent SE. Data have been evaluated by paired Student’s *t* test using GraphPad Prism 7 and a level of *P* < 0.05 was considered statistically significant.

## Supplementary information

Supplementary Figure 1

Supplementary Figure 2

Supplementary Figure 3

Supplementary Figure 4

Supplementary Figure 5

Supplementary Figure 6

Supplementary Figure 7

Supplementary Figure 8

Supplementary Figure 9

Supplementary Figure 10
