# Supplementary material for: The homeobox transcription factor MEIS2 is a regulator of cancer cell survival and IMiDs activity in Multiple Myeloma: modulation by Bromodomain and Extra-Terminal (BET) protein inhibitors
Source: Cell Death Dis. 2019 Apr 11;10(4):324. doi: 10.1038/s41419-019-1562-9 (PMC6459881; doi:10.1038/s41419-019-1562-9)
Supplement: Supplementary file 1 — Supplementary Figure 1 [file 41419_2019_1562_MOESM1_ESM.pdf]

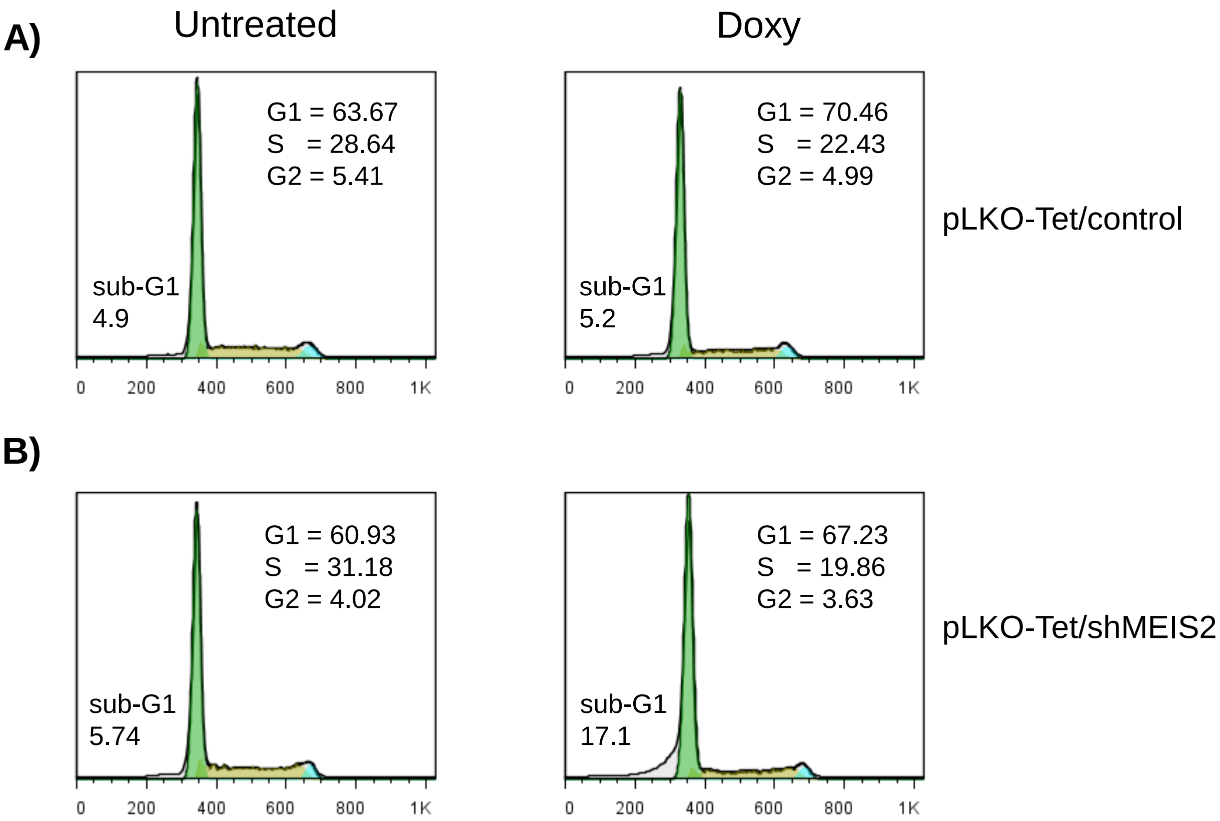

Suppl. Fig. 1 - SKO-007(J3)/shMEIS2-Tet cells were treated for 7 days with 100 ng/ml Doxycycline, then fixed and stained with PI to analyze cell distribution among the different cell-cycle phases, indicated in each panel. Data are representative of one out of three independent experiments.
