# Supplementary material for: The homeobox transcription factor MEIS2 is a regulator of cancer cell survival and IMiDs activity in Multiple Myeloma: modulation by Bromodomain and Extra-Terminal (BET) protein inhibitors
Source: Cell Death Dis. 2019 Apr 11;10(4):324. doi: 10.1038/s41419-019-1562-9 (PMC6459881; doi:10.1038/s41419-019-1562-9)
Supplement: Supplementary file 2 — Supplementary Figure 2 [file 41419_2019_1562_MOESM2_ESM.pdf]

A)

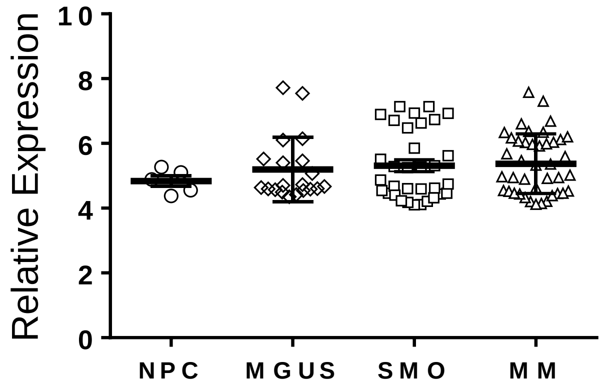

GSE47552 (GEO2R analysis)
NPC = 5
MGUS = 20
SMO = 33
MM = 41

B)

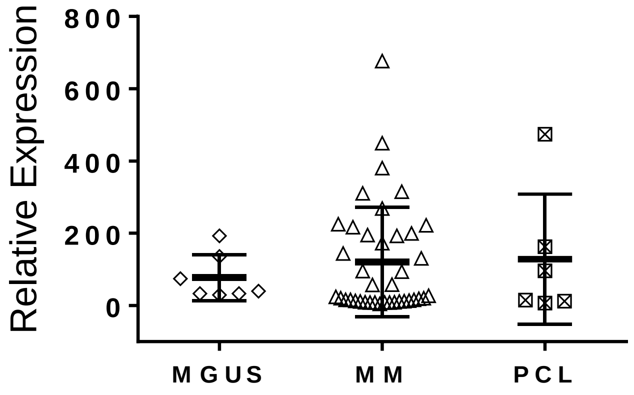

GSE2113 (GEO2R analysis)
MGUS = 7
MM = 39
PCL = 6

Suppl. Fig. 2 - Expression of MEIS2 and MM progression. A, B) Relative mRNA expression of MEIS2 (GEO2R analysis of the indicated microarray public data) in MM patients: normal B cells, normal PCs (NPC), MGUS, Smoldering, MM, and plasma cell leukemia (PCL).
