# Supplementary material for: The homeobox transcription factor MEIS2 is a regulator of cancer cell survival and IMiDs activity in Multiple Myeloma: modulation by Bromodomain and Extra-Terminal (BET) protein inhibitors
Source: Cell Death Dis. 2019 Apr 11;10(4):324. doi: 10.1038/s41419-019-1562-9 (PMC6459881; doi:10.1038/s41419-019-1562-9)
Supplement: Supplementary file 3 — Supplementary Figure 3 [file 41419_2019_1562_MOESM3_ESM.pdf]

Set size	Hits	Pvalue	Goid-Desc	Genes
76	48	4.9e-24	1: protein targeting to ER (7:11)	RPL35, CHMP4B, RPL36, RPL10A, PMM1, RPL3, RPL6, RPL7, RPL12, RPL15, RPL18, RPL19, RPL22, RPL24, RPL27, RPL29, RPL31, RPL32, RPL35A, RPL37, RPL38, RPL39, RPL36A, RPLP0, RPLP1, RPS3, RPS5, RPS6, RPS7, RPS9, RPS10, RPS15, RPS16, RPS17, RPS19, RPS21, RPS24, RPS25, RPS27A, RPS 28, SRP14, SRP19, SRP54, SRP68, SRP72, SSR3, UBA52, RPL14
102	50	8.8e-16	1: protein localization to endoplasmic reticulum (7:7)	KDELR1, RPL35, CHMP4B, RPL36, RPL10A, PMM1, RPL3, RPL6, RPL7, RPL12, RPL15, RPL18, RPL19, RPL22, RPL24, RPL27, RPL29, RPL31, RPL32, RPL35A, RPL37, RPL38, RPL39, RPL36A, RPLP0, RPLP1, RPS3, RPS5, RPS6, RPS7, RPS9, RPS10, RPS15, RPS16, RPS17, RPS19, RPS21, RPS24, RPS25, RPS27A, RPS28, SRP14, SRP19, SRP54, SRP68, SRP72, SSR3, UBA52, RPL14, PEX16
4595	919	3.0e-05	1: gene expression (5:5)	**over 100 entries** (2, 442, 475,)
1533	322	1.1e-04	1: negative regulation of gene expression (5:7)	**over 100 entries** (2, 165, 165,)
75	24	2.7e-03	1: intrinsic apoptotic signaling pathway by p53 class mediator (6:8)	TOPORS, CDKN1A, DDX5, FHIT, KDM1A, HIPK2, CDIP1, HINT1, ING2, MDM2, MSX1, RPS27L, TAF9B, PMAIP1, POU4F1, RPS7, PERP, ZMAT3, AEN, STK11, TP53BP2, TMEM109, DYRK2, ZMAT1
20	9	4.4e-03	1: mitochondrial electron transport, cytochrome c to oxygen (7:15)	COX5B, COX6B1, COX6C, COX7C, COX15, NDUFA4, CYCS, COX7A2L, COX5A
56	18	9.9e-03	1: cellular senescence (5:5)	CDK6, CDKN1A, CDKN2A, ULK3, RSL1D1, H2AFX, HMGA1, ING2, PRMT6, RBL1, SRF, TERF2, KAT6A, YPEL3, MAPKAPK5, PNPT1, EEF1E1, NUAK1

Suppl. Fig. 3 - MEIS2 modulates transcription of genes involved in ER homeostasis, gene expression, apoptosis, mitochondrial function or senescence. Gene Ontology enrichment (selected) over all the transcripts significantly correlated with MEIS2
