# Supplementary material for: The homeobox transcription factor MEIS2 is a regulator of cancer cell survival and IMiDs activity in Multiple Myeloma: modulation by Bromodomain and Extra-Terminal (BET) protein inhibitors
Source: Cell Death Dis. 2019 Apr 11;10(4):324. doi: 10.1038/s41419-019-1562-9 (PMC6459881; doi:10.1038/s41419-019-1562-9)
Supplement: Supplementary file 4 — Supplementary Figure 4 [file 41419_2019_1562_MOESM4_ESM.pdf]

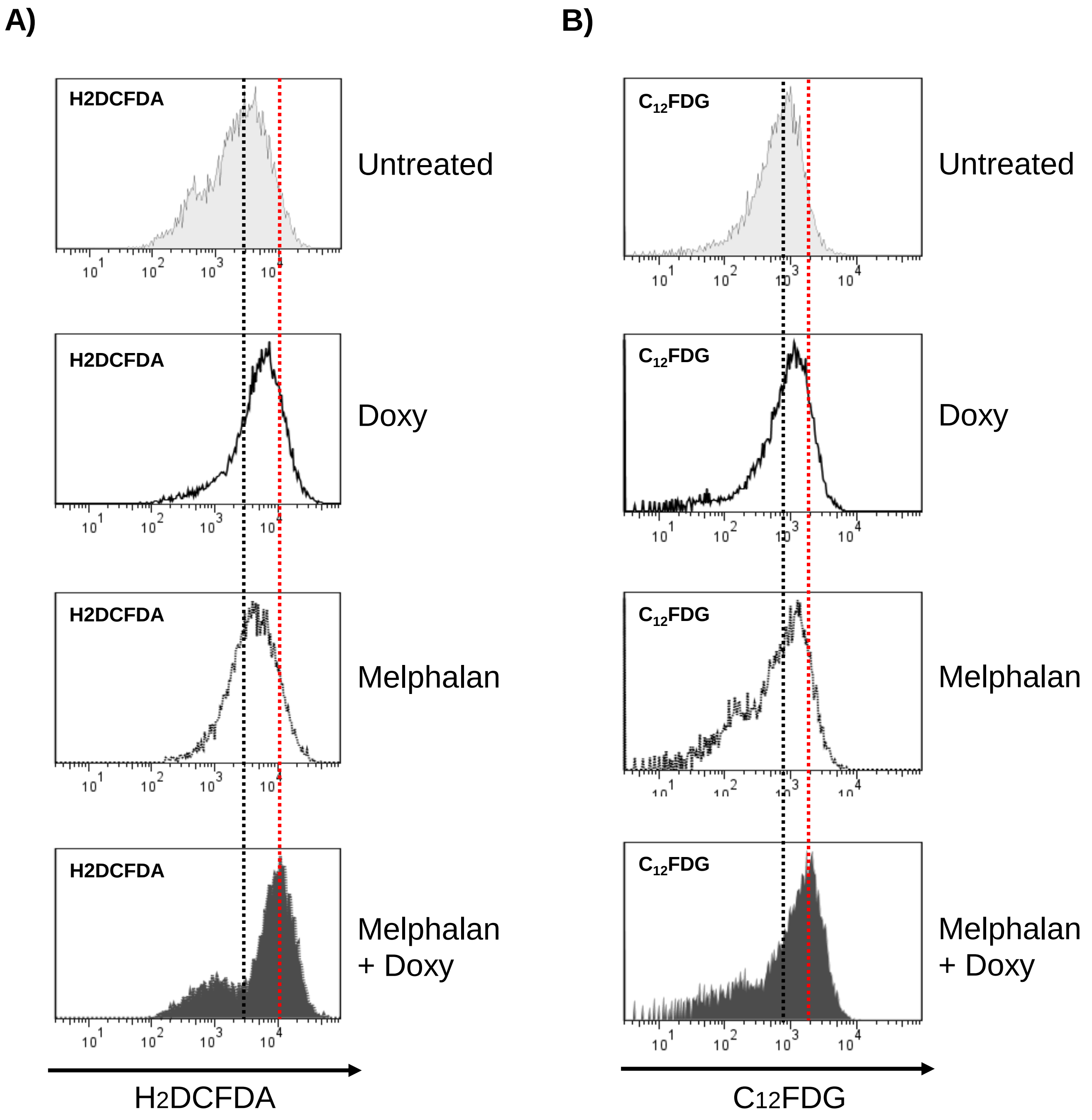

Suppl. Fig. 4 - MEIS2 regulates ROS production and senescence in response to Melphalan. A) Quantification of ROS production in SKO-007(J3)/shMEIS2-Tet cells, untreated or treated with 100 ng/ml Doxycycline for 72h and incubated with Melphalan for 24h as described above. Histograms represent the H_2DCFDA signal detected as FL-1 green fluorescence emission. Data are representative of one out of three independent experiments. B) Senescence Associated (SA)-βGal activity of SKO-007(J3)/shMEIS2-Tet cells treated as described above. SA-βGal assay was performed using the fluorogenic substrate C_{12}FDG to measure βGal activity by flow cytometry. Histograms represent the C_{12}-fluorescein signal detected as FL-1 green fluorescence emission.
