# Supplementary material for: The homeobox transcription factor MEIS2 is a regulator of cancer cell survival and IMiDs activity in Multiple Myeloma: modulation by Bromodomain and Extra-Terminal (BET) protein inhibitors
Source: Cell Death Dis. 2019 Apr 11;10(4):324. doi: 10.1038/s41419-019-1562-9 (PMC6459881; doi:10.1038/s41419-019-1562-9)
Supplement: Supplementary file 5 — Supplementary Figure 5 [file 41419_2019_1562_MOESM5_ESM.pdf]

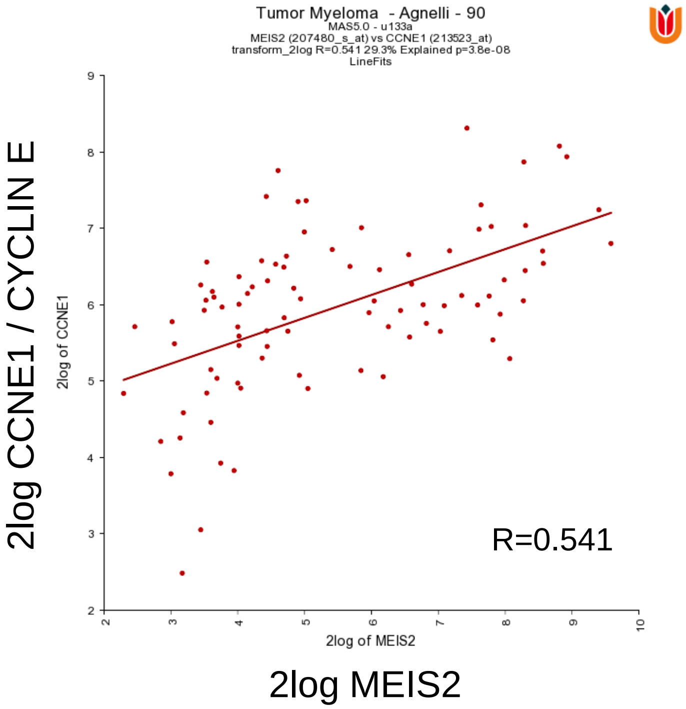

R2 Dataset
MM (Agnelli)

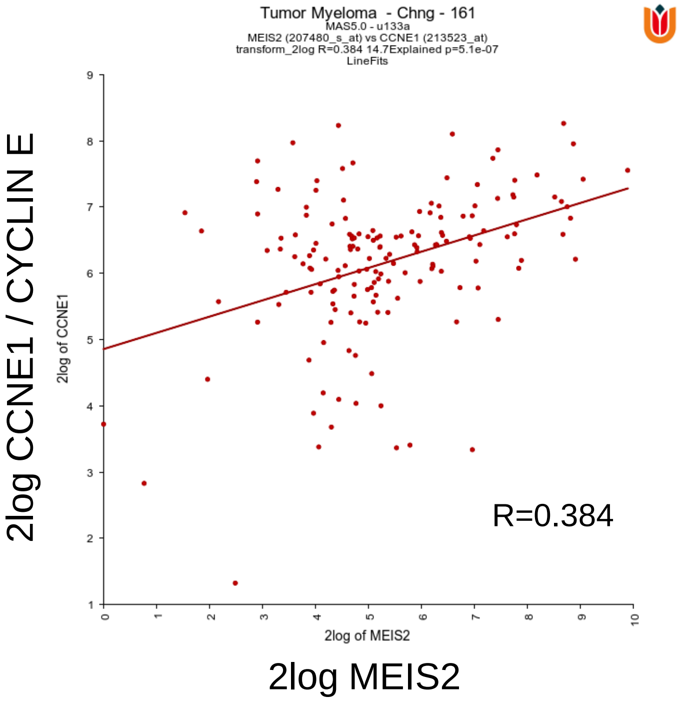

R2 Dataset
MM (Chng)

Suppl. Fig. 5 - Correlation between MEIS2 and Cyclin E/CCNE1 expression in MM. Correlation between MEIS2 and Cyclin E/CCNE1 expression (Agnelli MM Dataset of R2 - R=0.541, p-value=3.8e^{-08} and Chng MM Dataset of R2 - R=0.384, p-value=5.1e^{-07}.
