# Supplementary material for: The homeobox transcription factor MEIS2 is a regulator of cancer cell survival and IMiDs activity in Multiple Myeloma: modulation by Bromodomain and Extra-Terminal (BET) protein inhibitors
Source: Cell Death Dis. 2019 Apr 11;10(4):324. doi: 10.1038/s41419-019-1562-9 (PMC6459881; doi:10.1038/s41419-019-1562-9)
Supplement: Supplementary file 6 — Supplementary Figure 6 [file 41419_2019_1562_MOESM6_ESM.pdf]

A)

MM1.S
JQ1 0.5µM / 6h
GSE44929
(GEO2R analysis)

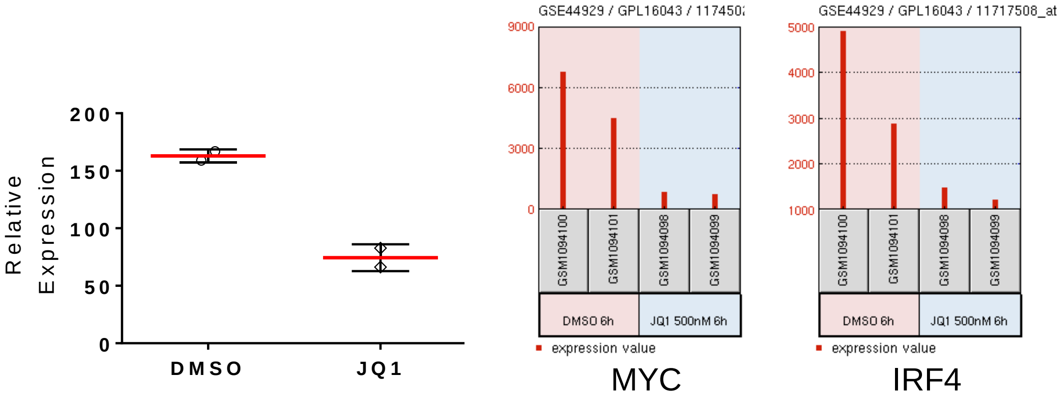

B)

MM1.S
JQ1 0.5µM / 24h
GSE31365
(GEO2R analysis)

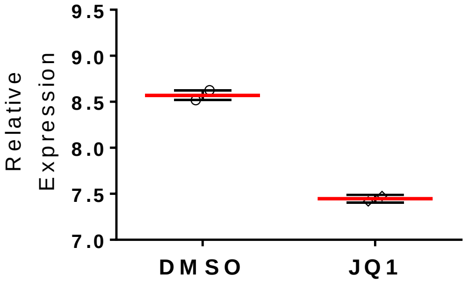

C)

KMS11
JQ1 0.5µM / 24h
GSE31365
(GEO2R analysis)

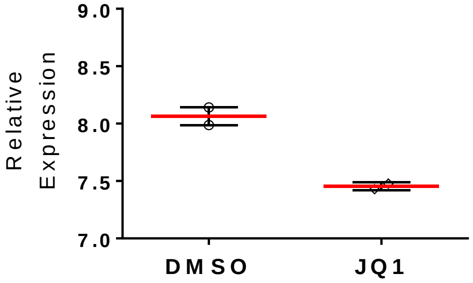

Suppl. Fig. 6 - BETi modulates MEIS2 in MM cell lines. A-C) Relative mRNA expression of MEIS2 (GEO2R analysis of the indicated microarray public data) in human MM cell lines. The indicated cell lines were treated with 0.5 µM JQ1 or DMSO for 6 or 24h. In panel A, the relative expression of MYC and IRF4 has been shown as positive controls for BETi activity in these cells.
