# Supplementary material for: The homeobox transcription factor MEIS2 is a regulator of cancer cell survival and IMiDs activity in Multiple Myeloma: modulation by Bromodomain and Extra-Terminal (BET) protein inhibitors
Source: Cell Death Dis. 2019 Apr 11;10(4):324. doi: 10.1038/s41419-019-1562-9 (PMC6459881; doi:10.1038/s41419-019-1562-9)
Supplement: Supplementary file 7 — Supplementary Figure 7 [file 41419_2019_1562_MOESM7_ESM.pdf]

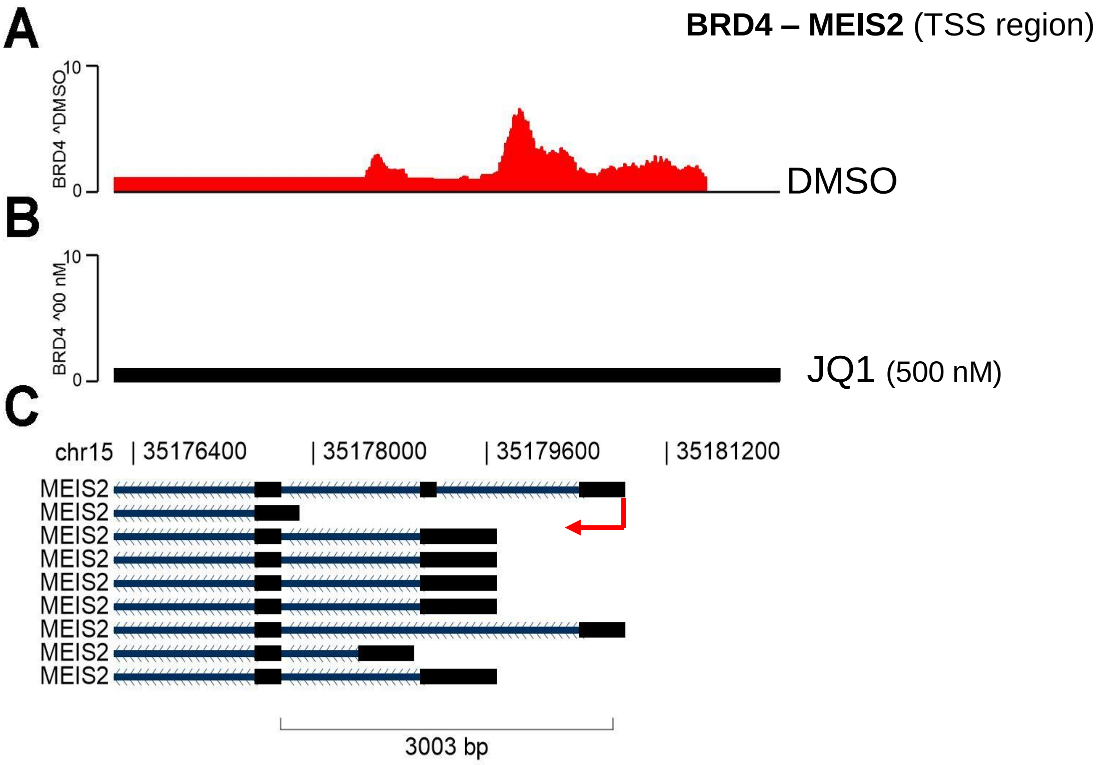

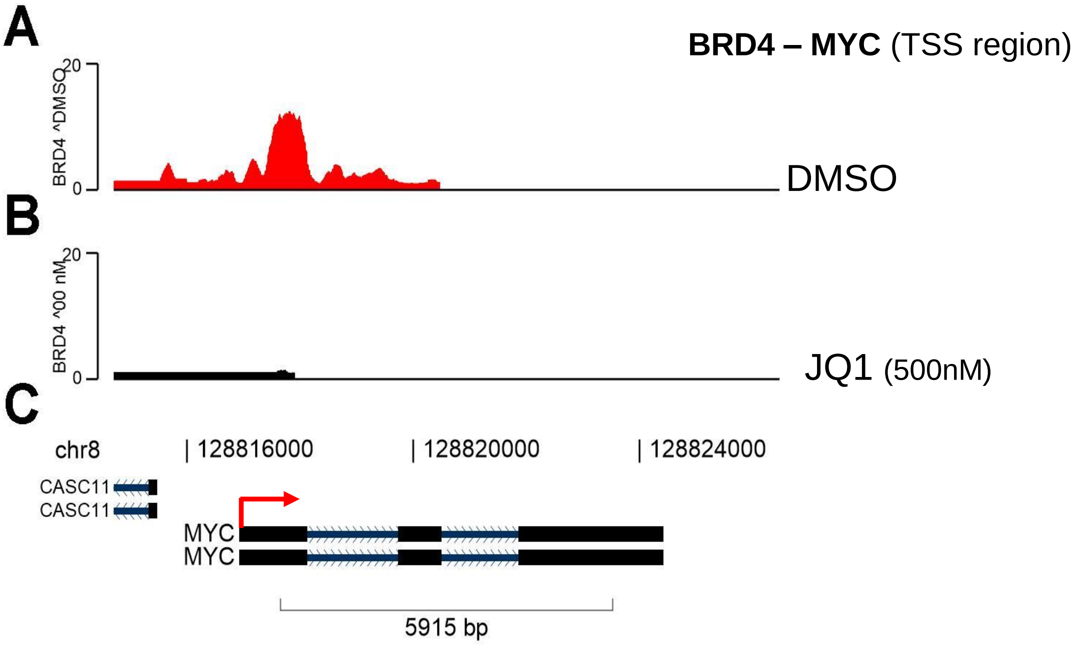

Suppl. Fig. 7 – A,B) BRD4 ChIP-Seq occupancy on MEIS2 and MYC (positive control) TSS regions. Analysis of aligned data associated with the GEO dataset accession ID GSE44931 (www.ncbi.nlm.nih.gov/geo/) (HG18) (MM1.S MM cells) and data visualization was performed and adapted using EaSeq Software (http://easeq.net). ChIP-seq signals in units of rpm/bp.
