# Supplementary material for: The homeobox transcription factor MEIS2 is a regulator of cancer cell survival and IMiDs activity in Multiple Myeloma: modulation by Bromodomain and Extra-Terminal (BET) protein inhibitors
Source: Cell Death Dis. 2019 Apr 11;10(4):324. doi: 10.1038/s41419-019-1562-9 (PMC6459881; doi:10.1038/s41419-019-1562-9)
Supplement: Supplementary file 8 — Supplementary Figure 8 [file 41419_2019_1562_MOESM8_ESM.pdf]

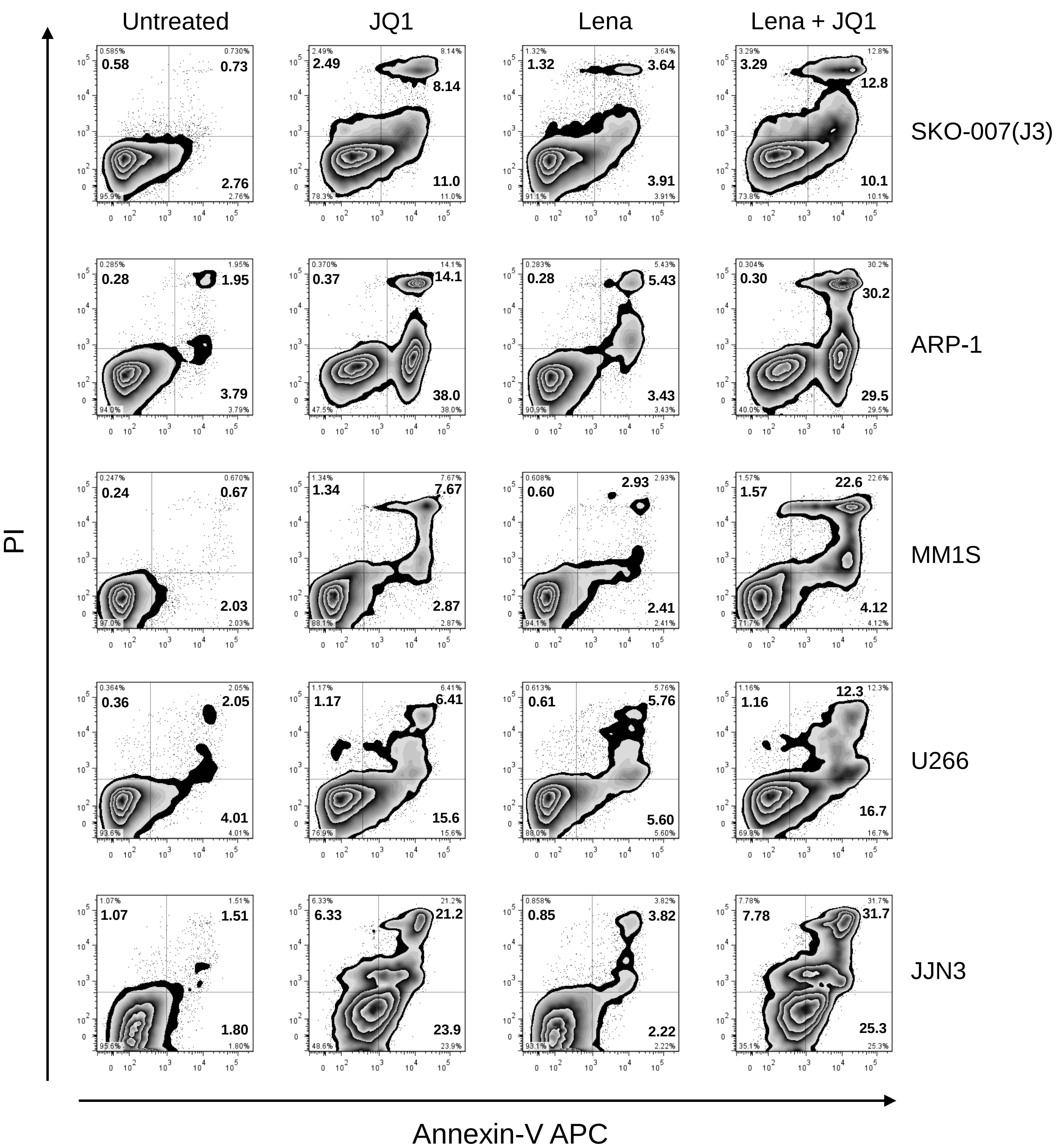

Suppl. Fig. 8 – **Combination of JQ1 and Lenalidomide increases apoptosis in MM cell lines.** Annexin-V assay: the indicated MM cell lines were treated with JQ1 0.5 μM, Lenalidomide 5 μM or the combination of these drugs, for 48h. Data are representative of one out of two independent experiments.
