# Supplementary material for: The homeobox transcription factor MEIS2 is a regulator of cancer cell survival and IMiDs activity in Multiple Myeloma: modulation by Bromodomain and Extra-Terminal (BET) protein inhibitors
Source: Cell Death Dis. 2019 Apr 11;10(4):324. doi: 10.1038/s41419-019-1562-9 (PMC6459881; doi:10.1038/s41419-019-1562-9)
Supplement: Supplementary file 9 — Supplementary Figure 9 [file 41419_2019_1562_MOESM9_ESM.pdf]

MYC mRNA

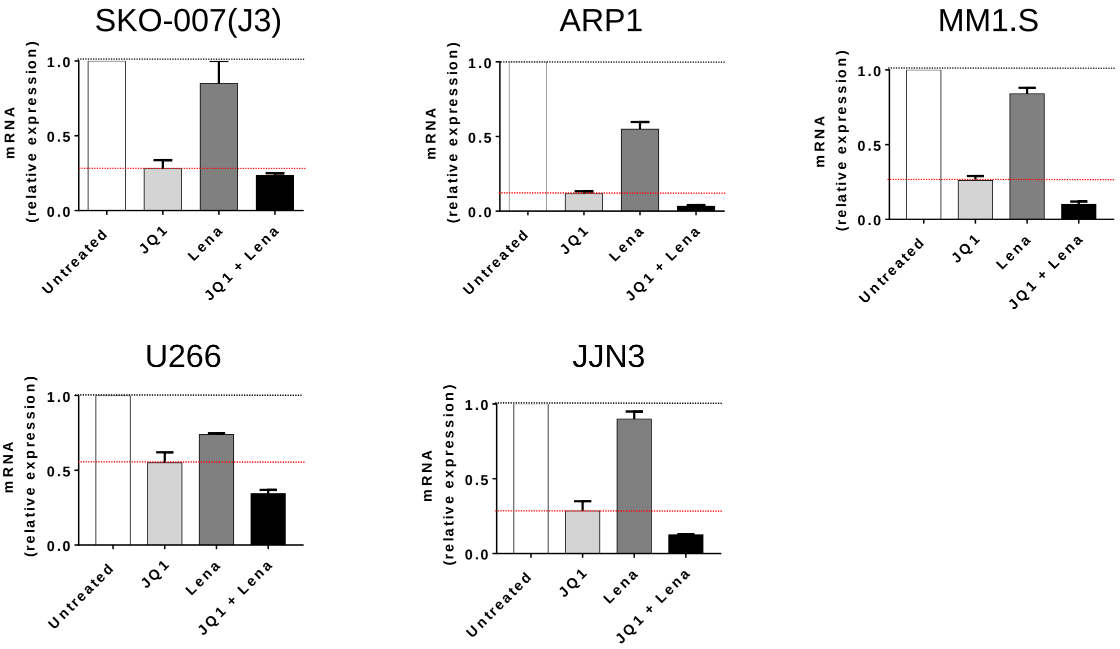

Suppl. Fig. 9 - Efficacy of JQ1 + Lenalidomide combination on MYC mRNA expression in MM cell lines. Total RNA was isolated from stimulated MM cells as indicated in the figure and analyzed by Real-Time qRT-PCR. Data, expressed as relative mRNA expression, were normalized with GAPDH and referred to the cells Untreated considered as calibrator and represent the mean ± SE of two independent experiments.
