# Supplementary material for: The homeobox transcription factor MEIS2 is a regulator of cancer cell survival and IMiDs activity in Multiple Myeloma: modulation by Bromodomain and Extra-Terminal (BET) protein inhibitors
Source: Cell Death Dis. 2019 Apr 11;10(4):324. doi: 10.1038/s41419-019-1562-9 (PMC6459881; doi:10.1038/s41419-019-1562-9)
Supplement: Supplementary file 10 — Supplementary Figure 10 [file 41419_2019_1562_MOESM10_ESM.pdf]

Patient no.	Sex/Age	Clinical Stage	Monoclonal Ig	% PCs in BM
1	F/72	Onset	IgG-k	30
2	M/62	Onset	IgG-ʎ	61
3	M/70	Relapse	IgG-k	41
4	M/71	Relapse	IgG-k	17

Suppl. Fig. 10 - Clinical parameters of MM patients.
